# Is lack of integrity a useful concept when dealing with health and social care pre-registration student fitness to practise cases?

**DOI:** 10.1177/0025817220947947

**Published:** 2020-09-17

**Authors:** Timothy J David, Sarah Ellson

**Affiliations:** 1 Faculty of Biology, Medicine and Health, University of Manchester, Manchester, UK; 2 UK Co-Head Regulatory, Fieldfisher LLP, Manchester, UK

**Keywords:** Integrity, dishonesty, fitness to practise, healthcare student

## Abstract

Health and social care regulators in their guidance to pre-registration students and registrants emphasise the importance of honesty and integrity. While the term honesty is generally understood, the meaning of integrity is less familiar, and for many years, there has been disagreement as to whether there is any difference between “dishonesty” and “lack of integrity.” To explore the possible application of lack of integrity to student behaviour, we present cases that illustrate what might be considered to demonstrate a lack of integrity. As with other allegations, if there is to be a finding of fact then an allegation of lack of integrity and its basis need to be clearly set out in advance of any hearing. If the term lack of integrity is to be useful, guidance from the regulators will need to explain the meaning of the term. If, however, agreement as to the meaning cannot be reached, maybe the term “integrity” should no longer be a standard accompaniment to the term “honesty.”

## Introduction

The meaning of the term dishonesty, and its application to the behaviour of professionals, which had been thought to be a settled matter, has been the subject of major recent change because of a Supreme Court case in 2018, *Ivey v Genting Casinos*.^1^ Removing the reference to the person’s own understanding of dishonesty, this set out the correct test when considering allegations of dishonesty:

- first, the tribunal or panel must determine the actual state of the individual’s knowledge or belief as to the facts; and
- second, whether this conduct was honest or dishonest is to be determined by applying the (objective) standards of ordinary decent people.

However, the meaning of the term “lack of integrity” has been far less clear, and there is debate about whether lack of integrity differs from dishonesty.

Health and social care regulators all require pre-registration health and social care students and registrants to “act with honesty and integrity” and to be “honest and trustworthy,” examples being the guidance for students and registrants from the General Medical Council (GMC).^2–4^ The GMC guidance on standards of behaviour for medical students^3^ helps to explain the meaning of the word “honesty” by giving nine examples of how to demonstrate honesty. GMC guidance on professional behaviour and fitness to practise (FTP) for medical students gives examples of dishonest behaviour, and it refers to conduct that might raise a question about a student’s honesty, trustworthiness or character.^4^ However, in keeping with the guidance from most health and social care regulators, the meaning of the word “integrity” is not explained or defined.

This paper briefly examines the meaning of the term “lack of integrity.” It uses examples of student FTP cases to help inform a discussion as to whether integrity or its absence are useful concepts when managing cases where student behaviour has caused concerns about a student’s professional suitability for their chosen future career.

## The meaning of integrity and lack of integrity – a legal controversy

The word “integrity” can be applied to an object or to human behaviour. In a medical context, lack of integrity when referring to an object could apply to sets of patients’ medical records from which all the operation notes and anaesthetic records had been removed for research purposes such as a study of a particular operation.

Regarding human behaviour, there has long been a controversy as to whether lack of integrity has the same meaning as dishonesty. In the USA, as far back as 1888, a court judgment defined the word “integrity” and concluded that its meaning was synonymous with “probity,” “honesty” and “uprightness.”^5^ In the UK, controversy has arisen in cases dealt with by the Financial Services Authority (FSA) and the Solicitors Regulation Authority (SRA). Some landmarks in the UK debate are as follows:

In *Hoodless and Blackwell v FSA *(2003), integrity was defined as: “In our view ‘integrity’ connotes moral soundness, rectitude and steady adherence to an ethical code. A person lacks integrity if unable to appreciate the distinction between what is honest or dishonest by ordinary standards.”^6^

However, many cautioned against trying to formulate a comprehensive test for integrity, and in *Vukelic v FSA* (2009), integrity was described as “a concept elusive to define in a vacuum but still readily recognisable by those with specialist knowledge and/or experience in a particular market.”^7^

A similar approach was adopted in cases before the Administrative Court involving solicitors. In *Chan v SRA* (2015), it was said, “In my view, it serves no purpose to expatiate on its meaning. Want of integrity is capable of being identified as present or not, as the case may be, by an informed tribunal or court by reference to the facts of a particular case.”^8^

In *Scott v SRA*, it was said that, “There is an obvious distinction between the two concepts …  A person can lack integrity without being dishonest.”^9^ One example which applied here was being reckless as to the use of various client accounts. As the Solicitors Disciplinary Tribunal found, “the appellant had not enquired as to the reasons for the improper payments and transfers out of client account; he had not cared at all about what he was instructed to authorise, and he had not shown any steady adherence to any kind of ethical code. Accordingly, it was not so much a case of what the appellant thought, but that he neither thought nor cared about what was required by the rules governing his profession, of which he was aware.”

In *Newell-Austin* *v SRA* (2017), it was concluded that regarding the meaning of lack of integrity:

- integrity connotes moral soundness, rectitude and steady adherence to an ethical code;
- no purpose is served by seeking to expatiate on the meaning of the term “lack of integrity”; and
- lack of integrity and dishonesty are not synonymous, and a person may lack integrity even though not established as dishonest.^10^

However, in a subsequent case of *Malins v SRA* (2017), it was concluded that definitions of honesty and integrity are aligned and synonymous.^11^

In *Wingate & Evans v SRA* (2018), it was concluded by the Court of Appeal^12^ that:

- the term “integrity” is a useful shorthand to express the higher standards expected from professional persons;
- it is impossible to formulate an all-purpose, comprehensive definition of integrity. But it is a counsel of despair to say: “Well you can always recognise it, but you can never describe it”;
- integrity connotes adherence to the ethical standards of one’s own profession. That involves more than mere honesty. A professional person is expected to be even more scrupulous about accuracy than a member of the general public; and
- the duty to act with integrity applies not only to what professional persons say, but also to what they do.

In *Adetoye v SRA* (2019), it was argued that if integrity denotes a higher moral standard than honesty, then it must surely follow that want of integrity is baser conduct than common-or-garden dishonesty. Mostyn J concluded, doing the best he could to reconcile the conflicting messages from the higher courts, that acting without integrity involves greater moral turpitude than mere dishonesty though paradoxically lack of integrity would generally attract a lesser sentence than dishonesty.^13^

## Guidance on integrity from the health and social care regulators

There are three main potential sources of guidance: guidance for registrants, guidance for students and guidance for tribunal members. Reference to integrity and lack of integrity is scanty compared to the guidance on dishonesty. A notable exception is the General Osteopathic Council and its guidance *Osteopathic practice standards*.^14^ Regarding lack of integrity, it provides these helpful examples in the final of four standards named “D1. You must act with honesty and integrity in your professional practice:

1. A lack of integrity in your practice can adversely affect patient care. Some examples are:
2. Putting your own interest above your duty to your patient;
3. Subjecting a patient to an investigation or treatment that is unnecessary or not in their best interests;
4. Deliberately withholding a necessary investigation, treatment or referral;
5. Prolonging treatment unnecessarily;
6. Putting pressure on a patient to obtain other professional advice or to purchase a product;
7. Recommending a professional service or product solely for financial gain;
8. Borrowing money from patients or accepting any other benefit that brings you financial gain.”

## A discussion of some student FTP cases

The cases referred to above involve financial matters, but these are rarely relevant in health and social care student FTP cases, which are usually concerned with other aspects of behaviour. We present brief outlines of some student FTP cases altered to ensure anonymity, with a short commentary on each, asking whether the described behaviour could or should be described as showing a lack of integrity.

### Medical student accessing famous patient’s medical records

A medical student used their NHS Trust ID to access the hospital medical records of a famous person in the national headlines having been admitted to hospital with a major surgical emergency.

**Commentary:** It is unclear from this description whether the student could be taken to task for misuse of their Trust ID, and the case description provides no information about the NHS Trust’s policies regarding accessing patient medical records and patient confidentiality. There must nevertheless be concern about a future health professional who accesses medical records purely to satisfy a curiosity about a famous person. Could one argue that this student displayed a lack of integrity?

### Nursing student repeatedly asks mentors to not document concerns

A nursing student displayed numerous problems with attendance, punctuality, communication, attitude and resistance to support, advice and warnings. Whenever attention was drawn to problems, the student would tell her mentors (supervisors) to not document or report concerns, asking for them to be overlooked, saying (with no explanation) this would be beneficial to her.

**Commentary:** The professional duty to keep careful student records applies just as much to educators as it does to health professionals. The student appears to be driven by a desire to avoid action being taken by the education providers. Could one argue this student’s repeated requests to conceal concerns about her behaviour should raise concerns about her integrity?

### Medical student trying to create an unfavourable impression of another student

Student 1, one of a pair of clinical students, was eager to favourably impress a consultant surgeon supervisor by creating an unfavourable impression of student 2. To do so, student 1 concealed the results of laboratory tests, so that when student 2 presented cases and reported to the supervisor that test results were not yet available, student 1 would proudly produce the missing results. This led to the supervisor telling student 2 to be more organised and efficient and reporting her to the medical school. However, a ward sister had spotted what was going on, and an investigation indicated that student 1 had been repeatedly concealing the results of tests to create a bad impression of student 2.

**Commentary:** A medical student successfully deceived a consultant supervisor and created an unfavourable impression of a fellow student. Many would regard concealing laboratory results as dishonest, but should one argue that, in addition, trying to harm the reputation of a professional colleague displayed a lack of integrity?

### A persistently uncooperative dental student

A dental student who experienced repeated atypical seizures (thought by several observers who had seen the attacks to have been faked) while on placement was referred to Occupational Health, which requested an independent psychiatric opinion. The student persistently refused to co-operate, repeatedly saying that his refusal was on the recommendation of his legal advisers. Much later, it transpired that the “legal advisers” were a single unqualified student friend who had attended law and ethics seminars. The student later admitted that he knew that the friend was not a lawyer but argued that he nevertheless believed that he had received sound legal advice and that he had not been trying to mislead.

**Commentary:** The student’s conduct probably did not justify a finding of dishonesty, but could one argue that creating a false impression that the student had been acting on the advice of one or more qualified lawyers showed a lack of integrity?

### A medical student who used his father to assess and certify a clinical skill

The certification of certain clinical skills is a GMC requirement for all UK medical students. A medical school regulation was that close family members could not supervise or assess students. A medical student used his father, a histopathologist working in another part of the UK, to assess and certify his abilities to insert an intravenous cannula, use an ophthalmoscope and obtain consent for a procedure. The three assessment forms were countersigned by the father.

**Commentary:** The use of a close family member had been contrary to the medical school regulations. However, in terms of patient safety, and the implications of ignoring the regulations, and selecting a doctor who by his clinical practice was unlikely to be familiar with the three clinical skills, should the student’s behaviour raise concerns about his integrity? If the motive in using the father was simply convenience that would be one matter, but if (not the case in this example) there had been evidence that the motive had been to select a doctor unlikely to have the necessary clinical skills, then a question of dishonesty would arise.

### Failure to inform NHS professionals of an investigation of alleged dishonesty

A final year student midwife faced multiple allegations of dishonesty – alleged falsification of signatures of supervisors confirming the completion of learning objectives and alleged falsification of attendance registers. Her studies had been suspended pending consideration of her case by the university FTP Committee. For some years, she had been employed in her spare time as a nursing assistant in a care home. She had considered declaring her referral to the FTP Committee to the care home, but her contract of employment did not indicate a requirement to declare an investigation of this sort nor did the university regulations require this, and she decided not to seek advice from the university or to inform her employers.

**Comments:** There was no evidence she had breached any specific university regulations. But particularly given that the employment was in the provision of health care, and given she was a final year student, might one conclude that her failure to inform her employer of her suspension from her studies and referral to the FTP Committee had shown a lack of integrity?

## Discussion

The examples presented here indicate that lack of integrity is not confined to financial matters and is a possible description of the behaviour of some health and social care students and professionals. The question is whether it might be useful to recognise and allege a lack of integrity, whether as distinct from dishonesty or as a feature in a case of dishonesty, and alongside other breaches of the code of conduct and/or regulations which apply to the student.

In some of these cases, there is a potential overlap between dishonesty and lack of integrity. In theory, it is possible to exhibit both characteristics either separately or in relation to the same matter. Could one argue that although dishonesty is a serious form of behaviour, separately recognising a lack of integrity could help a student better to understand the error of their behaviour? Or would it be more appropriate to view dishonesty and lack of integrity as being part of the same spectrum of behaviours, with a varying ability to distinguish between the two? However, in some of these examples, such as the student who accessed the medical records of a famous person, or the nursing student working at a care home, a question of dishonesty did not arise.

If lack of integrity is to be useful when tackling problem behaviours in student or registrant health and social care professionals, then this would need to be included in any list of allegations. As a basic principle, as with other findings of fact, it would not be possible for a committee considering FTP to make a finding of fact of a lack of integrity unless before the hearing the student or registrant fully knows the allegation and the reasons that underpin it.

Further, if lack of integrity is to be a useful concept in managing problem behaviours, then its meaning will need to be explained and included in guidance on expected professional behaviour. Although the term integrity is often coupled with that of honesty, when referring to desirable behaviour, there is a notable dearth of such guidance. The inability of the judiciary to formulate a comprehensive definition of integrity helps to illustrate the difficulties that lie ahead, and it may help to explain why the regulators provide so little by way of explanation. A recent Court of Appeal judgment (*Wingate and Evans v SRA* (2018))^12^ illustrated what might constitute a lack of integrity by a solicitor:

- a sole practice giving the appearance of being a partnership and deliberately flouting the conduct rules;
- recklessly, but not dishonestly, allowing a court to be misled;
- subordinating the interests of the clients to the solicitors’ own financial interests;
- making improper payments out of the client account;
- allowing the firm of solicitors to become involved in conveyancing transactions which bear the hallmarks of mortgage fraud; and
- making false representations on behalf of the client.

As set out in this judgment, “The duty of integrity does not require professional people to be paragons of virtue. In every instance, professional integrity is linked to the manner in which that particular profession professes to serve the public.”

If it proves impossible to provide an agreed definition and explanation of the term integrity, should its use be removed from professional guidance?

Finally, despite the lack of a clear and agreed definition, integrity has emerged as a front-runner amongst the desirable attributes to select applicants for admission to a medical school, there being significant associations between an integrity-based situational judgment test and other desirable personality traits.^15^ A situational judgment test that measured integrity was administered to 402 prospective applicants to the Rotterdam Medical School.^16^ This consisted of 57 scenarios, each of which was followed by four response options. Scenarios that assessed the ability to recognise *inappropriate* responses (i.e. what one should *not* do) appeared to have a validity greater than the ability to recognise *appropriate* responses (i.e. what one *should* do), which was thought to be due to a greater consensus on what is considered inappropriate than what is considered appropriate in a challenging situation.
